# Bioactive nanoglass regulating the myogenic differentiation and skeletal muscle regeneration

**DOI:** 10.1093/rb/rbad059

**Published:** 2023-06-07

**Authors:** Dagogo Dorothy Winston, Ting Li, Bo Lei

**Affiliations:** Key Laboratory of Shaanxi Province for Craniofacial Precision Medicine Research, College of Stomatology, Frontier Institute of Science and Technology, Xi'an Jiaotong University, Xi'an 710000, China; Key Laboratory of Shaanxi Province for Craniofacial Precision Medicine Research, College of Stomatology, Frontier Institute of Science and Technology, Xi'an Jiaotong University, Xi'an 710000, China; Key Laboratory of Shaanxi Province for Craniofacial Precision Medicine Research, College of Stomatology, Frontier Institute of Science and Technology, Xi'an Jiaotong University, Xi'an 710000, China; Xi'an Jiaotong University, Xi'an 710000, China; State-Key Laboratory for Mechanical Behavior of Materials, Xi’an Jiaotong University, Xi’an 710054, China

**Keywords:** bioactive materials, bioactive ceramic, bioactive glass nanoparticles, skeletal tissue engineering

## Abstract

Bioactive glass nanoparticles (BGNs) are widely used in the field of biomedicine, including drug delivery, gene therapy, tumor therapy, bioimaging, molecular markers and tissue engineering. Researchers are interested in using BGNs in bone, heart and skin regeneration. However, there is inadequate information on skeletal muscle tissue engineering, limited information on the biological effects of BGNs on myoblasts, and the role of bioactive glass composite materials on myogenic differentiation is unknown. Herein, we report the effects of BGNs with different compositions (60Si-BGN, 80Si-BGN, 100Si-BGN) on the myogenic differentiation in C2C12 cells and *in vivo* skeletal tissue regeneration. The results showed that 80Si-BGN could efficiently promote the myogenic differentiation of C1C12 cells, including the myotube formation and myogenic gene expression. The *in vivo* experiment in a rat skeletal muscle defect model also confirmed that 80Si-BGN could significantly improve the complete regeneration of skeletal muscle tissue during 4 weeks implantation. This work firstly demonstrated evidence that BGN could be the bioactive material in enhancing skeletal muscle regeneration.

## Graphical Abstract

**Figure:**
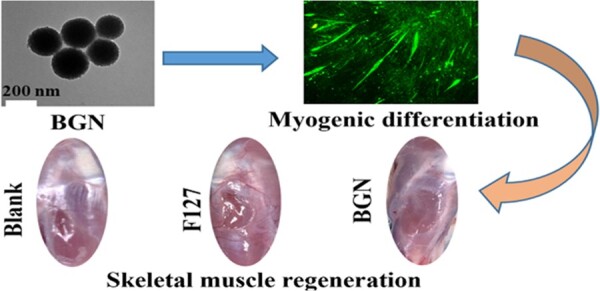

## Introduction

Bioactive glass (BG), originally, as developed in 1969 by Professor Hench, was composed of SiO_2_-CaO-Na_2_O-P_2_O_5_, which has been used in tissue regeneration, especially bone tissue regeneration [1]. There have been a number of efforts to dope this system with additional medicinal chemicals or to reformulate it in order to increase its biomedical uses. BGs have the unique bioactivity of adhering to live tissue and promoting new tissue development when exposed to physiological fluids [2, 3]. As the third-generation biomaterial owing to its biocompatibility and bioactivity, BGs have shown promising applications in tissue engineering [4]. For example, previous study has reported a therapeutically regenerative nano-hybrid (BSr@PPE) based on the bioactive Si-Ca-Sr nanoglass with graded versatility to overcome the challenges of tumor and infection injury repair [5]. There are several biological uses for bioactive glass nanoparticles (BGNs) due to their tiny size, high surface area-to-volume ratio, and high specific surface area, wide range of applications and their morphological properties are particularly appealing [6]. Since the shape and content of BGN are so largely dependent on the synthesis process, using BGNs in biological applications is only viable if the synthesis process is regulated. Melt-quenching and sol-gel procedures may be used to synthesize BGN, as well as other methods. A flame spray method has been used to make BGNs over the last decade [7]. A flame reactor is used to melt proportional precursors, which are subsequently quenched to produce BGNs. Alternatively, micron-sized BGs created from melt may be milled to produce BGNs [8]. While melt-quenching-based methods allow for the large-scale synthesis of BGNs, it is difficult to manage the BGNs' properties. In addition, the shape and size distribution of these BGNs are often irregular. It is difficult to use melt-quenching procedures for BGN synthesis in large-scale manufacturing since the equipment is so complicated. As an alternative, the sol-gel technique may be used to create BGNs with a more controlled form and size [9–13].

To present, BGNs have been extensively studied in a variety of biomedical applications, including as drug delivery, gene therapy, tumor treatment, bioimaging and tissue engineering [14–18]. For tissue repair and tissue engineering, BGNs have drawn considerable interest in the areas of bone, skin and dental tissue regeneration [19–24]. In recent years, few studies report the effect of silicate BG biomaterials on the skeletal muscle repair [25, 26]. Previous studies showed that silicate 45S5 BG and borate BG could enhance the skeletal muscle regeneration, mesoporous BGNs could increase the myotube formation of C2C12 myoblasts [25, 26]. However, no detailed studies on the effect of BGN with different compositions on the myoblast differentiation and *in vivo* skeletal muscle regeneration were reported. Herein, we want to investigate the effect of BGNs with different compositions on C2C12 murine myoblast proliferation and differentiation, as well as the *in vivo* skeletal muscle regeneration.

## Materials and methods

### Synthesis and characterizations of BGNs

According to the previous report, we synthesized BGN NPs by using a standard sol-gel co-template method [21]. The pure silica (SiO_2_), 80SiO_2_16CaO4P_2_O_5_ and 60SiO_2_-36CaO-4P_2_O_5_ were denoted as 100Si-BGN, 80Si-BGN and 60Si-BGN respectively (molar percent). The morphology of BGN was studied by the transmission electron microscope (TEM, HT-7700, Hitachi), and the acceleration voltage was 100 kV. The phase and chemical structure of BGN were analyzed by X-ray diffraction (DMAX/A, Rigaku) and Fourier transform infrared spectroscopy (FTIR, Nicolet 6700, Thermo Scientific). Elemental analysis of BGN was performed by field emission scanning electron microscopy (FESEM, Quanta™250 FEG) (FEI, Eindhoven, the Netherlands) combined with energy dispersion spectroscopy (EDS, Oxford Instruments X-Max).

### Cell culture

The myoblast C2C12 was bought from ATCC (American Type Culture Collection). The fibroblast L929 was obtained from National Collection of Authenticated Cell Cultures. Complete medium (GM) for C2C12 and L929 cells consisted of DMEM medium plus 10% fetal bovine serum and 1% penicillin. The differentiated medium (DM) for C2C12 cells were DMEM medium contained 2% Hestrin–Schramm (HS) and 1% penicillin for cell proliferation and differentiation studies. Cells were cultured in a cell incubator (Thermo Fisher) at 37°C, 5% CO_2_ and 100% humidity.

### Cytotoxicity evaluation

C2C12 and L929 cells were planted in a 96-well cell culture plate (CostarTM, Corning) at a seeding density of 5000 cells/well to examine the cytotoxicity of BGNs. BGNs at varying doses (50, 100, 150, 200 μg/ml) were added to the culture media after the cells were adhered on the base of the multi-well culture plates. The cytotoxicity tests were carried out using the AlamarBlue cell viability kit and the LIVE/DEAD kit (Invitrogen). Cell survival and stained fluorescent morphology were examined using a fluorescence microscope (Olympus IX53) and a microplate reader (molecular devices) at 560/600 nm after 1, 3 and 5 days of co-cultivation. There were six replicates in this experiment (*n* = 6).

### Myogenic differentiation investigations

At a density of 50 000 cells per well, the C2C12 cells were used for myogenic differentiation assessment. After cells were cultured in GM medium for 24 h for cell apposition, the culture medium was changed to DM supplemented with various concentrations of BGNs (ranging from 0 to 100 μg/ml), and the medium was changed every other day. The real-time quantitative polymerase chain reaction (RT-PCR) assessments were carried out after the immunofluorescence staining had been carried out for preset amounts of time in the culture.

### Immunofluorescence staining

In order to examine the expression of myosin heavy chain (MHC) protein in the myogenic differentiation of C2C12 cells after growth in DM containing BGNs, immunofluorescence labeling was performed. After 7 days of culture, the cells were thoroughly cleaned three times with phosphate-buffered saline (PBS), fixed on 2.5% glutaraldehyde for 0.5 h, and infiltrated with 0.1% Triton X-100. After washing, non-specific binding was prevented by incubation with 1% bovine serum albumin (BSA, Sigma) at 37°C for 1 h. Subsequently, C2C12 cells were washed and placed in culture medium containing mouse anti-MHC monoclonal antibody (1:1000 dilution) and incubated at 4°C for 12 h. After the wells were cleaned, secondary Alexa Flour 488 Goat Anti-Rabbit IgG (H L)(1:500, molecular probe) was placed into each well and incubated at 37°C for another 2 h. After cleaning with PBS, the cells were treated with 0.1% 4′,6-diamidino-2-phenylindole (DAPI) for 10 min and then examined using inverted fluorescence microscopy. The number of muscle tubes, their length and diameter, and their maturity index were extracted from at least three images from each sample using the program ImageJ.

### RT-PCR analysis

After being cultivated with BGNs in DM for 7 days, C2C12 cells were subjected to RT-PCR in order to determine the levels of myogenic gene expression of MHC and Myogenin (MyoG). At intervals of time that were previously defined, total RNA from the C2C12 cells was extracted. Utilizing the Transcriptor First Strand cDNA Synthesis Kit, the cDNA synthesis was successfully completed (Roche). RT-PCR was carried out using a 7500 Fast real-time PCR System (Applied Biosystems) with SYBR Select Master mix (Invitrogen) serving as the master mix and primer sequences for MHC and MyoG (All in-One TM qPCR Primer, GeneCopoeia) serving as the primers (40 cycles, melting at 95°C for 3 s, annealing and extension at 60°C for 15 s). The comparative threshold cycle (Ct) approach was used in order to conduct the analysis of the RT-PCR investigation's data. GAPDH was the gene responsible for housekeeping (primer 5′-AGGTCGGTGTGAACGGATTTG-3′ and 5′-TGTAGACCATGTAGTTGAGGTCA-3′). Three separate analyses were performed on each sample.

### Rat model of skeletal muscle injury

Skeletal muscle regeneration was studied using the rat tibialis anterior muscle defect [27, 28]. In this study, SD rats (female, 180–200 g) were anesthetized with isoflurane, with six rats in each experimental group. The tibialis anterior muscle was exposed by making an anterolateral skin incision. With dimensions of 5 mm in length by 3 mm in breadth and depth, the muscular belly was left with an abnormally large cuboid-shaped defect. Thermal-responsive nanocomposite hydrogels (F127-BGN) were prepared using Pluronic F127 (F127, a linear ABA triblock copolymer) in combination with BGN to fill muscle defects. The content of BGN in F127 hydrogel was 40 μg/ml. Then F127-BGN was injected into the muscle defect to produce nanocomposite hydrogels at the wound site (37°C). A sham surgery was used as a control, as was a pure hydrogel of F127. After that, an absorbable surgical suture was used to close up the wound on the skin (Ethicon). Throughout the studies, no significant immunological, infectious, or dyskinetic effects were found. In accordance with Xi'an Jiaotong University's Institutional Animal Ethics Committee's approval (No. 2019-1167), the research was carried out on animals in accordance with all applicable institutional and national regulations for the care of laboratory animals.

### Histochemical staining

In order to investigate the processes of repair and regeneration in skeletal muscle tissue, the histochemical staining was used [29]. In skeletal muscle defects, measurements were taken of the myofiber diameter, the number of centronucleated myofibers and the capillary density. The nuclei of new skeletal muscle cells are located in the middle. At each time point (1 and 4 weeks), the regenerated tissue was removed and fixed with 10% formalin. Following this, regenerated tissues were removed and fixated with 10% formalin. After the trimming process was complete, the skeletal muscle tissues were immersed and fixed in paraffin. Using a microtome, slices with a thickness of 5 μm were then cut from the paraffin block. The sections were stained using the hematoxylin and eosin (H–E) staining kit according to the manufacturer's instructions (Beyotime). H–E-stained sections were used to conduct the measurements that determined the myofiber diameter, the total number of centronucleated myofibers and the capillary density. Using an Olympus optical microscope, three randomly chosen fields from each section and three sections from each specimen were reviewed, and the digitized information was processed using the Image J program.

### Statistical analysis

All the data were expressed as mean ± standard deviation. Using the GraphPad Prism 6 (La Jolla) software, the data were statistically analyzed by a one-way ANOVA test. Differences were considered statistically significant when *P <* *0.05 and **P <* *0.05.

## Results and discussion

### Structure characterization of BGNs

**Figure 1.**
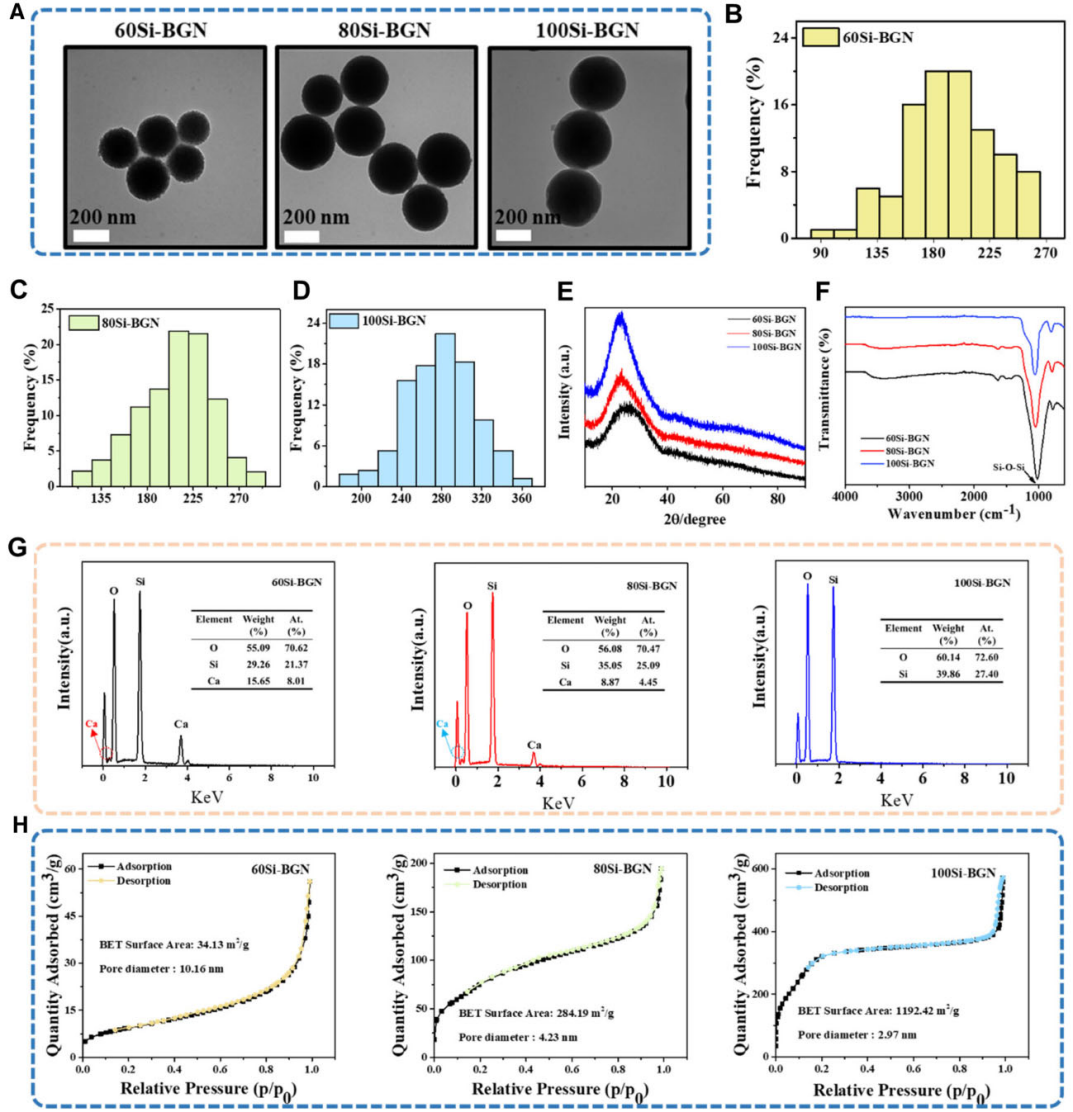
**The physicochemical structure characterization of BGNs.** (**A**) The TEM images of 60Si-BGN, 80Si-BGN, 100Si-BGN. Size distribution of 60Si-BGN (**B**), 80Si-BGN (**C**) and 100Si-BGN (**D**) based on the TEM images. FTIR (**E**) and XRD (**F**) analyses of 60Si-BGN, 80Si-BGN and 100Si-BGN. (**G**) EDS spectrum and element percentage of 60Si-BGN, 80Si-BGN and 100Si-BGN. (**H**) N2 adsorption–desorption isotherms of 60Si-BGN, 80Si-BGN and 100Si-BGN.

The synthesis and structure characterizations of BGNs were performed and the results are shown in Fig. 1.

The TEM images of BGNs showed that 60Si-BGN, 80Si-BGN and 100Si-BGN were the spherical morphology, and the surface aperture of BGNs decreases with the increase of Si content (Fig. 1A). The statistical results showed that the particle size of 60Si-BGN was 160–200 nm, that of 80Si-BGN was 180–220 nm and that of 100Si-BGN was 240–300 nm (Fig. 1B–D), suggesting the decrease of Si content reduced the particle size of nanoparticles. The XRD patterns of 60Si-BGN, 80Si-BGN and 100Si-BGN showed the absence of sharp diffraction peaks, which proved that the synthesized BGN of three different components are all amorphous structures (Fig. 1E). The FTIR spectra showed the intense peaks at 1000–1100 cm^−1^ which was attributed to the symmetrical stretching mode Si-O-Si in 60Si-BGN, 80Si-BGN and 100Si-BGN (Fig. 1F). Figure 1G shows the EDS spectra of samples, in which the typical Si and Ca peaks could be found in 60Si-BGN and 80Si-BGN. Additionally, no obvious Ca peak was observed in the EDS spectra in 100Si-BGN (the pure SiO_2_). The specific surface areas of 60Si-BGN, 80Si-BGN and 100Si-BGN were measured and the mesoporous structures were estimated by multi-point Brumul-Emmett-Taylor (BET) N_2_ absorption method. The specific surface area increased with the increase of Si content and the pore size decreased with the increase of Si content (Fig. 1H). The average pore sizes of 60Si-BGN, 80Si-BGN and 100Si-BGN are 2.97, 4.23 and 10.16 nm, respectively. In the next work, we investigated the effect of 60Si-BGN, 80Si-BGN and 100Si-BGN on the behavior of myogenic cells.

### Cytotoxicity evaluation of BGNs

**Figure 2.**
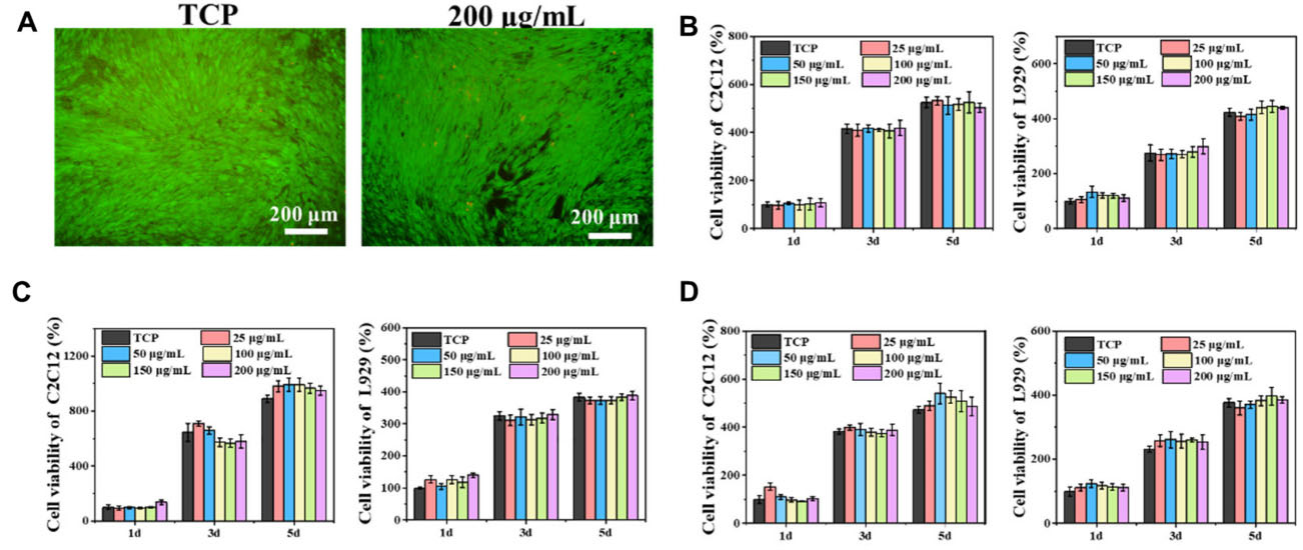
Cytotoxicity analysis of as-prepared BGNs. (**A**) LIVE/DEAD stained fluorescent pictures of C1C12 cells after culture with 80Si-BGN at 200 μg/ml; cell viability of C2C12 and L929 after culture with 60Si-BGN (**B**), 80Si-BGN (**C**) and 100Si-BGN (**D**) at different concentrations. Tissue culture plate (TCP) was used as a control. All experiments were performed in triplicate (*n* = 6, *P <* *0.05 and **P <* *0.01 compare with TCP).

For cell viability and toxicity testing, LIVE/DEAD analysis was utilized. C2C12 cells were co-cultured with 0–200 μg/ml 80Si-BGN for 5 days. The quite many green live cells and a small number of red dead cells are visible, and the cell has a good condition and growth, the C2C12 cells spread over the entire field of the view (Fig. 2A). For cell viability testing, the C2C12 cells line and L929 cell line were co-cultured with 60Si-BGN, 80Si-BGN and 100Si-BGN at the concentration ranges from 0 to 200 μg/ml for 1, 3 and 5 days, cell viability was investigated and detected by AlamarBlue™ kit (Fig. 2B–D). On the first day of co-cultivation, the cell viability in each concentration range was no significant difference than that of the control group, namely the tissue culture plate (TCP) group. On the 5th day of co-cultivation, the cell viability did not increase significantly compared with that on the 3rd day. This may be due to the rapid proliferation of C2C12, and L929 cell line, the cells quickly reached 100% confluence, and there was not enough growth space in the 96-well culture plate. In general, the results of this experiment showed that 60Si-BGN, 80Si-BGN and 100Si-BGN had good cell compatibility with C2C12 and L929 cell line. However, after cultured for 3 days, the 80Si-BGN with different concentrations significantly enhanced the proliferation of C2C12 and L929 cells, compared with other BGNs. The possible reason probably that the Ca content in BGN has the different effect on the cell activity. The small amount of calcium can promote cell activity and the high calcium content would reduce this effect [30]. The 80Si-BGN has the suitable calcium content and therefore could enhance the cell activity.

### Myogenic differentiation analysis

**Figure 3.**
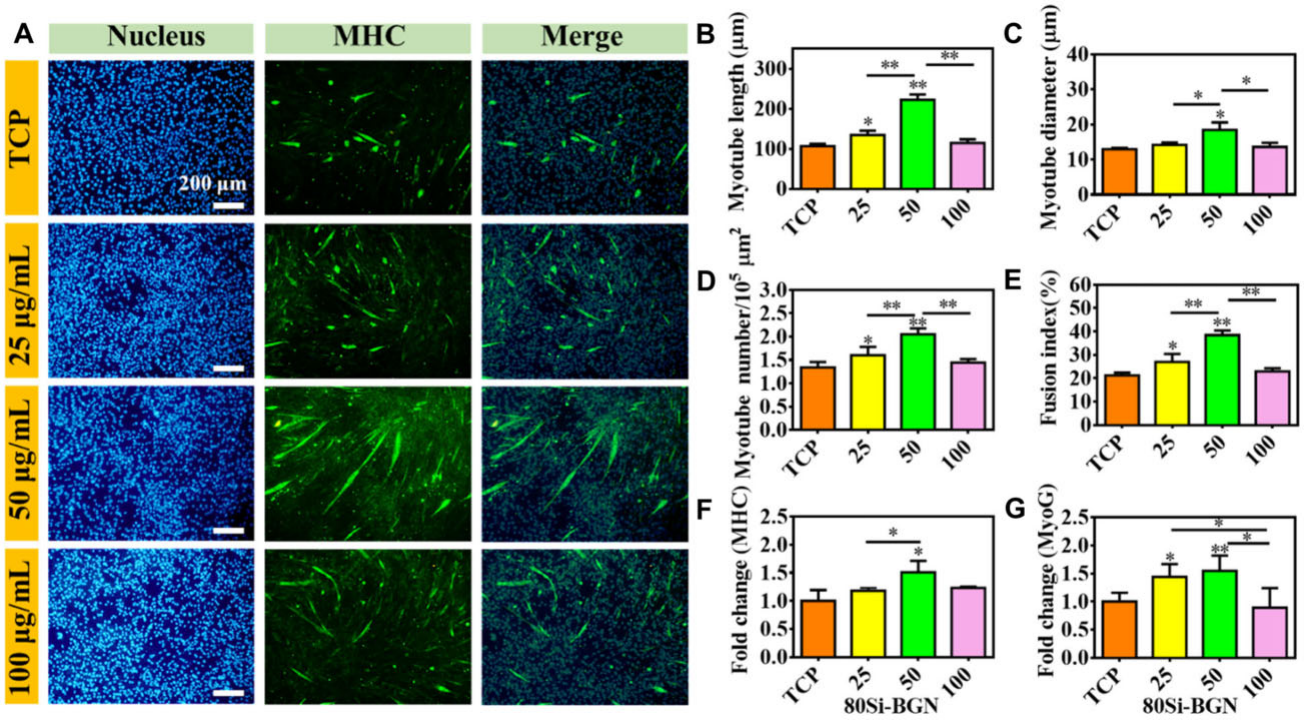
Myogenic differentiation evaluation of C2C12 cells after culture with 80Si-BGN for 7 days. (**A**) Immunofluorescent staining images showing the nucleus (blue), MHC protein (green) in C1C12 cells after culture with nanoparticles at different concentrations; calculated results on the myotube length (**B**), myotube diameter (**C**), myotube number (**D**) and fusion index (**E**); and fold change MHC gene (**F**) and MyoG gene (**G**). All experiments were performed in triplicate (*n* = 3, *P <* *0.05 and **P <* *0.01 compare with TCP).

**Figure 4.**
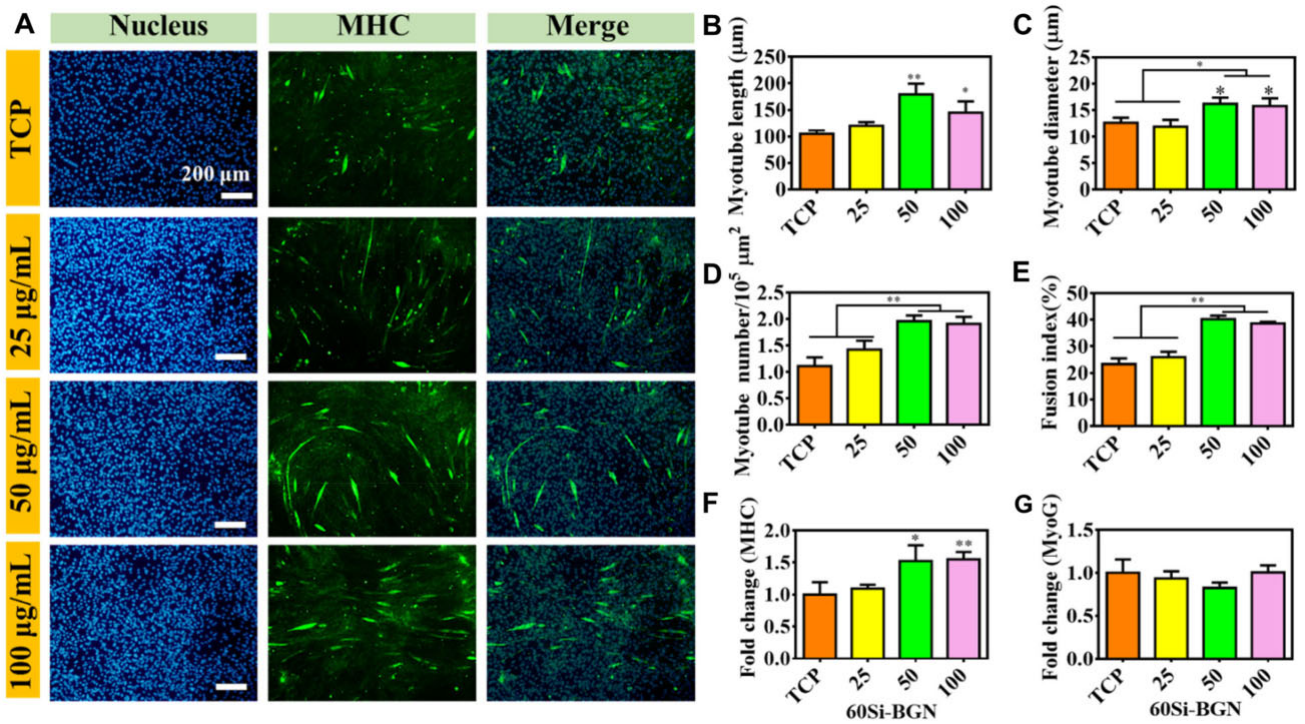
Myogenic differentiation evaluation of C2C12 cells after culture with 60Si-BGN for 7 days. (**A**) Immunofluorescent staining images showing the nucleus (blue), MHC protein (green) in C1C12 cells after culture with nanoparticles at different concentrations; calculated results on the myotube length (**B**), myotube diameter (**C**), myotube number (**D**) and fusion index (**E**); fold change MHC gene (**F**) and MyoG gene (**G**). All experiments were performed in triplicate (*n* = 3, *P <* *0.05 and **P <* *0.01 compare with TCP).

**Figure 5.**
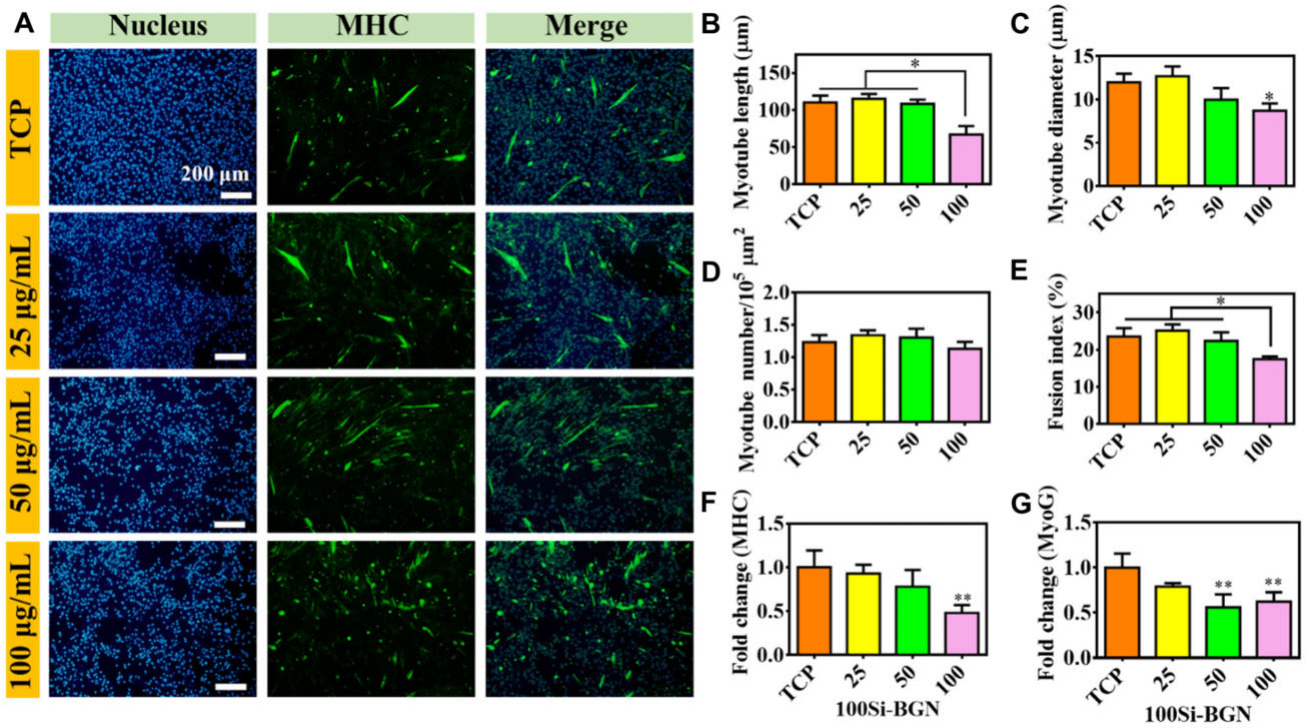
Myogenic differentiation evaluation of C2C12 cells after culture with 100Si-BGN for 7 days. (**A**) Immunofluorescent staining images showing the nucleus (blue), MHC protein (green) in C1C12 cells after culture with nanoparticles at different concentrations; calculated results on the myotube length (**B**), myotube diameter (**C**), myotube number (**D**) and fusion index (**E**); and fold change MHC gene (**F**) and MyoG gene (**G**). All experiments were performed in triplicate (*n* = 3, *P <* *0.05 and **P <* *0.01 compare with TCP).

During the whole differentiation stages of myogenic differentiation, the MHC protein is highly expressed [31]. The multinucleated myotubes formation in C2C12 myoblasts could be shown through the immunofluorescent staining of MHC protein. The immunofluorescent staining could stain the cells as the green fluorescence when the C1C12 cells were fused to be the numerous nuclei morphology. The cellular immunofluorescent morphology and the number of central karyotype newborn skeletal muscle cells in each group of defects was counted, and the results are shown in Figs. 3–5.

Figure 3 shows the effect of 80Si-BGN at different concentrations on the myotube formation of C2C12 cells after culture for 7 days. The immunofluorescent staining of MHC protein showed that the myotube morphology formation was significantly dependent on the content of 80Si-BGN (Fig. 3A). There were no obvious myotube formation (green) in TCP group, and the green fluorescent cells were increased significantly when the content of 80Si-BGN was increased at 50 μg/ml. At the same time, it was found that there are multiple blue nuclei in the differentiated myotubes, and the number of nuclei implies and reflects the maturity of myotubes. In addition, the myotube length, myotube diameter, myotube number and fusion index in C2C12 cells after culture with 80Si-BGN indicated that 80Si-BGN with the 50 μg/ml demonstrated the significantly high results as compared to TCP and other groups (Fig. 3B–E). In this study, the mRNA expression of MyoG and MHC genes related to myogenic differentiation was detected to reflect the differentiation of C2C12 cells in the early, middle and late stages, as shown in Fig. 3F–G. Under the condition of complete differentiation medium, when C2C12 cells reach enough confluence and much confluence between the cells, myogenic differentiation and cell fusion may also occur. As shown in Fig. 3F, the mRNA expression levels of MHC genes in C2C12 cells in 80Si-BGN at 50 μg/ml were significantly higher than the TCP group and 25 μg/ml group. The changes in results of the MyoG gene were similar with the MHC in Fig. 3F. In a word, the 80Si-BGN at 50 μg/ml could sufficiently stimulate the myotube formation and myogenic genes expressions of C2C12 cells. Although the results showed that the 80 Si-BGN at 50 μg/ml could increase the myotube information and have no effect on the myotube formation at 100 μg/ml, compared with TCP control, we believe this would not affect their *in vivo* therapeutic application because we could inject suitable nanoparticles content in the tissue defect and show the therapeutic role. The high content of nanoparticles did not affect the myotube formation.

Figure 4 shows the effect of 60Si-BGN at different concentrations on the myotube formation of C2C12 cells after culture for 7 days. The immunofluorescent staining of MHC protein showed that the myotube morphology formation was significantly dependent on the content of 60Si-BGN (Fig. 4A). The TCP control group showed that there was no obvious green myotube morphology, suggesting the poor myotube formation in TCP group. After culture with 60Si-BGN at different concentrations (25–100 μg/ml) for 7 days, the obvious myotube formation could be observed. The calculated results showed that the significantly high myotube length, myotube diameter, myotube number and fusion index in C2C12 cells could be found in 50 μg/ml group and 100 μg/ml group (Fig. 4B–E). For the myogenic gene expressions, the results were also related with the contents with 60Si-BGN (Fig. 4F and G). There was significantly high MHC gene expression in 50 μg/ml group and 100 μg/ml group, as compared with TCP group and 25 μg/ml group (Fig. 4F). However, there was no significant difference on the MyoG gene expression between different groups (Fig. 4G). These results showed that 60Si-BGN may enhance the myogenic differentiation of C2C12 cells through the activation of MHC gene and protein.

To demonstrate the difference of myogenic differentiation between BGN with different compositions, the effect of 100Si-BGN on the C2C12 differentiation was also investigated, as shown in Fig. 5. The MHC protein fluorescent images showed that there was no obvious change on the green fluorescence staining after 7 days of culture (Fig. 5A). Different with 60Si-BGN and 80Si-BGN, the myotube length, myotube diameter, myotube number and fusion index were very similar among different groups, and high 100Si-BGN concentration (100 μg/ml) even inhibited the myotube length, myotube diameter and fusion index (Fig. 5B–E). For the MHC gene expression, the 100Si-BGN with 25 μg/ml group and 50 μg/ml showed no significant effect, and the high 100 μg/ml inhibited the gene expression (Fig. 5F). The performance of MyoG gene was very similar with the MHC gene (Fig. 5G). When the content of 100Si-BGN was 50 and 100 μg/ml, the expression of MyoG was significantly inhibited, compared with TCP and 25 μg/ml group.

The results on the myogenic differentiation in C2C12 cells showed that the chemical composition of BGN has the significant effect on the myotube formation and myogenic gene expressions. The 80Si-BGN showed the significantly high capacity on enhancing the myogenic differentiation, compared to the 60 Si-BGN and 100Si-BGN. The 60Si-BGN, 80Si-BGN and 100Si-BGN possessed the chemical structure of 60SiO_2_-36CaO-4P_2_O_5_, 80SiO_2_-16CaO-4P_2_O_5_ and 100SiO_2_ respectively. At the same concentrations of nanoparticles, the high silica content (100Si) significantly inhibited the myogenic differentiation (100Si-BGN). The results of 60Si-BGN and 80Si-BGN showed that the presence of Ca in the glass network significantly enhanced the myogenic differentiation of nanoparticles. As compared to the 60Si-BGN, the 80Si-BGN presented the better results on the myogenic differentiation, which suggested that the content of Ca also had the effect on the myogenic differentiation. The suitable ratio of Si-Ca such as 5:1 (80Si-BGN) may have the positive capacity on enhancing the myogenic differentiation in C2C12 cells. Calcium signaling plays an important role in the development, maintenance and regeneration of skeletal muscle, and reducing intracellular calcium levels can inhibit the differentiation of C2C12 myoblasts [32, 33]. After uptaken by myoblasts, the synthesized BGN may promote the differentiation of myoblasts by increasing intracellular calcium levels by releasing calcium ions. Our results indirectly demonstrated that silica-doped BGN may promote myoblast differentiation and skeletal muscle regeneration through calcium signaling pathways by regulating intracellular calcium levels to release calcium ions. The further studies would be carried out for demonstrating the potential mechanism of BGN on promoting the myogenic differentiation.

### Skeletal muscle tissue regeneration *in vivo*

Based on the *in vitro* results, it was very interesting to investigate if the 80Si-BGN could enhance the *in vivo* skeletal muscle regeneration. Therefore, in this study, a skeletal muscle defect with a length of 5 mm, a width of 3 mm and a depth of 2 mm was made in the middle of the tibialis anterior muscle of the rat to construct a large-volume muscle loss model of the tibialis anterior muscle of the rat.

**Figure 6.**
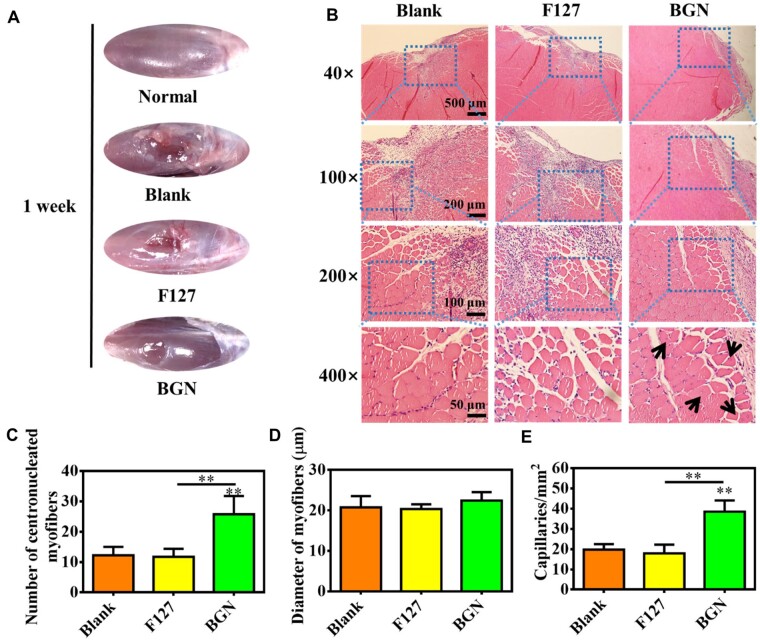
*In vivo* repair evaluation of tibialis anterior muscle defect in rats after 1 week. (**A**) Apparent observation on the skeletal tissue repair after 1 week; (**B**) H–E sections staining images of muscle tissue after treating with different samples after 1 week; and calculated results on the number of centronucleated myofibers (**C**), diameter of myofibers (**D**) and density of capillaries (**E**). **P <* *0.01.

Figure 6 shows the skeletal muscle repair results after implanting the 80Si-BGN hydrogel for 1 week. The skeletal muscle defect in blank control group was still very visible and that in F127 control group showed a little repair (Fig. 6A). Compared with blank and F127 control groups, the significant repair on the skeletal muscle defect in 80Si-BGN group could be observed. Figure 6B presents the H–E-stained images of skeletal muscle tissue after repair by various biomaterials. The tissue section staining showed that the new muscle tissue formation could be seen in the 80Si-BGN group, compared with blank and F127 group. Additionally, in the enlarged images, the 80Si-BGN group indicated the significant myofiber morphology with a centronucleated character (Fig. 6B). The calculated results further showed that there was significantly high number of centronucleated myofiber in the 80Si-BGN group compared with blank and F127 group, as shown in Fig. 6C. There was no significant difference on the diameter of myofiber in various groups (Fig. 6D). To show the effect of BGN on the angiogenesis during the skeletal muscle regeneration, the density of capillaries in the tissue sections was calculated. As shown in Fig. 6E, the density of capillaries in 80Si-BGN group was significantly higher than those in blank and F127 group. These results demonstrated that 80Si-BGN could significantly enhance the skeletal muscle tissue formation through improving the myofiber and angiogenesis after 1 week.

**Figure 7.**
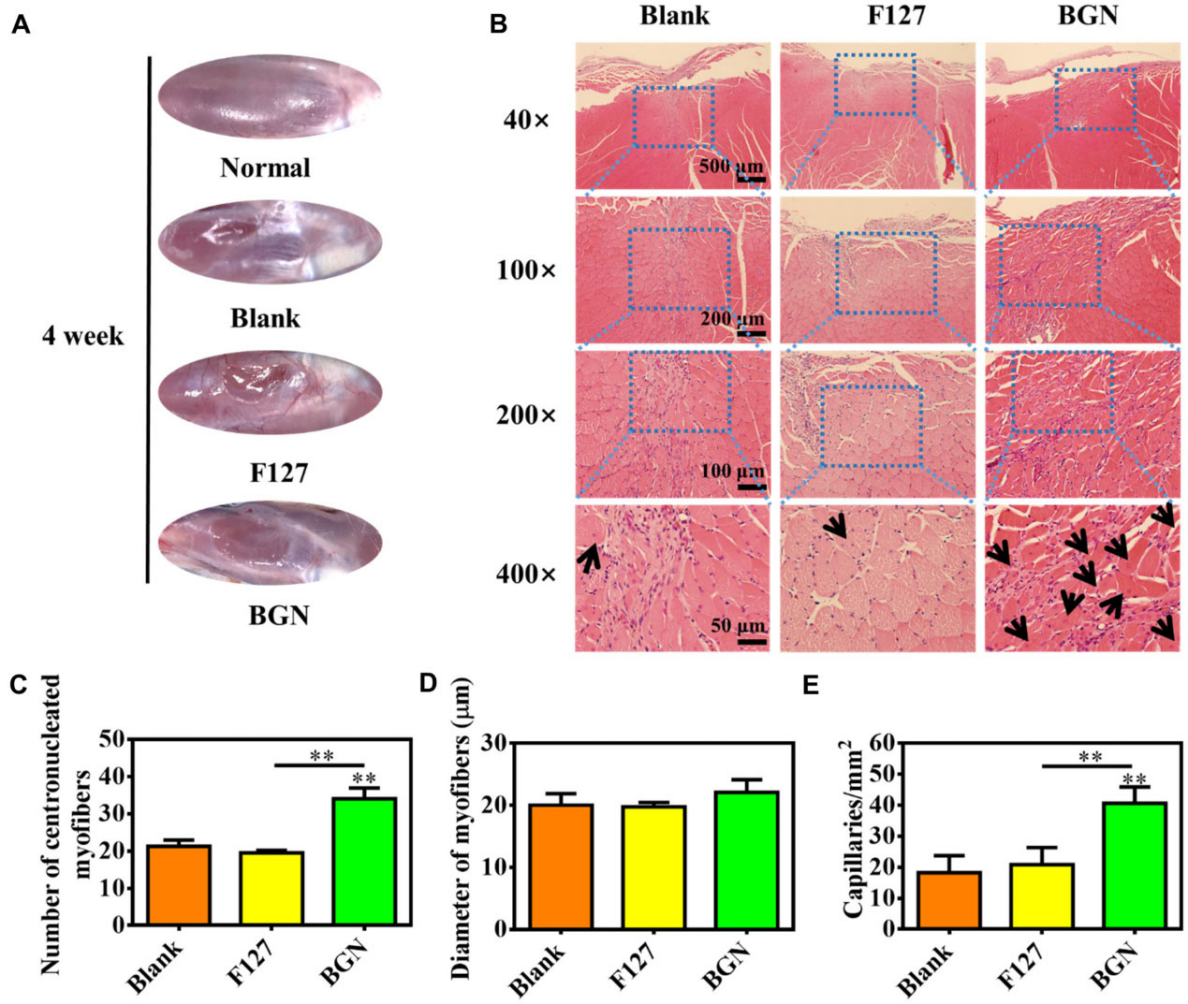
*In vivo* repair evaluation of tibialis anterior muscle defect in rats after 4 weeks. (**A**) Apparent observation on the skeletal tissue repair after 4 weeks; (**B**) H–E sections staining images of muscle tissue after treating with different samples after 4 weeks; and calculated results on the number of centronucleated myofibers (**C**), diameter of myofibers (**D**) and density of capillaries (**E**). **P <* *0.01.

Figure 7 shows the skeletal muscle repair results after implanting the 80Si-BGN hydrogel for 4 weeks. After the 4th week of skeletal muscle defect repair, the skeletal muscle defects in each group became shallower, but it can be clearly seen that the blank control group and the F127 group are still markedly recessed (Fig. 7A). The skeletal muscle defect in the 80Si-BGN group has recovered well, but the new tissue indicates that there are still boundaries between the mouse skeletal muscles, were smooth enough. In the 80Si-BGN group, the regenerated skeletal muscle tissue was smooth, and the depression disappeared. The further H–E-stained images showed the new muscle tissue formation after treating with different biomaterials (Fig. 7B). The results showed that the skeletal muscle defect has been filled with the new born muscle tissue, and the significantly good result could be seen in 80Si-BGN treated group compared with other groups. Different from normal adult skeletal muscle cells, in newborn skeletal muscle cells, the nucleus is in the center of the cell, rather than at the periphery near the cell membrane. According to the results of H–E staining of skeletal muscle sections, the number of central karyotype newborn skeletal muscle cells in each group of defects was counted, and the results are shown in Fig. 7C–E. The statistical results of the above illustration found that the number of central karyotype neonatal skeletal muscle cells in the 80Si-BGN group was significantly higher than that in the F127 and blank groups (Fig. 7C). No significant difference on the diameter of myofiber formed in the tissue between different groups was observed (Fig. 7D). In addition to the number of new skeletal muscle cells, new blood vessels are also an important indicator for evaluating tissue repair. Blood vessels provide tissues with nutrients and oxygen, and take away metabolites, which are the basic guarantee for maintaining the normal function of the tissues. The number of new blood vessels in the defect of the 80Si-BGN group was significantly higher than that in the F127 and blank group (Fig. 7E). The increase in new blood vessels is beneficial to the repair and reconstruction of skeletal muscle defects.

The experimental results of *in vivo* repair of the tibial anterior muscle defect in rats showed that 80Si-BGN can increase the number of skeletal muscle cells and the number of new blood vessels in the central karyotype of the defect, and promote the healing of the defect, which is beneficial to the large endogenous regeneration of mouse skeletal muscle defects. Animal experiments further confirmed the application potential of BGN NPs in skeletal muscle tissue engineering. Although this study clearly confirmed that 80Si-BGN has great potential for treating skeletal muscle defect, the detailed molecular mechanism on regulating myogenic differentiation should be investigated clearly.

## Conclusion

In summary, we prepared BGNs with different compositions including 60Si-BGN, 80Si-BGN, 100Si-BGN and found the effect of BGN on the myogenic differentiation and skeletal muscle tissue regeneration. The 60Si-BGN and 80Si-BGN could significantly enhance the myogenic differentiation and skeletal muscle tissue regeneration, especially 80Si-BGN. The 100Si-BGN showed the negative results on regulating the myoblast differentiation. This work showed that the suitable silicon and calcium ratio played an important role in improving the myogenic differentiation and skeletal muscle regeneration.
